# Neurogenic rosacea could be a small fiber neuropathy

**DOI:** 10.3389/fpain.2023.1122134

**Published:** 2023-02-20

**Authors:** Min Li, Meng Tao, Yue Zhang, Ruoxin Pan, Duoduo Gu, Yang Xu

**Affiliations:** Department of Dermatology, The First Affiliated Hospital of Nanjing Medical University, Nanjing, China

**Keywords:** dysesthesia, neurogenic rosacea, small fiber neuropathy, diagnostic criteria, treatment

Small fiber neuropathy (SFN) is a peripheral neuropathy of the small myelinated Aδ fibers and unmyelinated C-fibers, affecting not only pain and temperature sensation but also autonomic system function. There are various SFN clinical manifestations, the most common being pain, dysesthesia, and dysautonomia. In recent literature, pruritus occurred in 68.3% of patients with SFN (1). Moreover, SFN is associated with a number of underlying diseases including metabolic, toxic, inflammatory, and autoimmune diseases, but remain idiopathic in approximately 40% of cases (2). The exact pathophysiological mechanism of SFN is complex. In the case of autoimmune etiology, damage to thin Aδ and C-fibers may be cytokine-mediated *via* tumor necrosis factor (TNF)-α, interleukin (IL)-2, IL-6, and IL-8 (3).

Diagnostic tools for SFN detection include skin biopsy with intraepidermal nerve fiber density (IENFD), quantitative sensory testing (QST), quantitative sudomotor axon reflex testing, corneal confocal microscopy, electromyography, and nerve-conduction studies that quantify and assess small nerve fiber function (4). SFN diagnosis is determined primarily by history and physical examination; however, functional neurophysiologic testing and skin biopsy evaluation of intraepidermal nerve fiber density can further confirm diagnosis.

Pain is a common and crucial SFN manifestation; thus, therapy is based on the underlying cause and pain management. Several classes of medications are commonly used, including antidepressants, anticonvulsants, opioids, and topical treatments. In previous studies, tricyclic antidepressants (TCAs), serotonin–norepinephrine reuptake inhibitors (SNRIs), and anticonvulsants such as gabapentin and pregabalin, were routinely recommended for neuropathic pain treatment (4).

Rosacea, a chronic inflammatory skin condition, primarily manifests as recurrent flushing and erythema in the central facial area. Rosacea can be divided into four subtypes: erythematotelangiectatic, papulopustular, phymatous, and ocular. Neurogenic rosacea (NR), a new variant suggested by Scharschmidt in 2011, is characterized by facial burning, stinging, and dysesthesia disproportionate to flushing, which manifested as more severe neurogenic inflammation and recalcitrant to traditional therapy (5). The disease severity of NR especially can be evaluated by QST, part of SFN's diagnostic criteria, is rare used in diagnosis of rosacea (6).

Innate immune system and neurovascular dysregulation play major roles in neurogenic rosacea pathophysiology. TNF-α expression, a known mediator of mast cell degranulation and vasodilation, is upregulated in patients with rosacea, promoting release of inflammatory mediators such as IL-6 and IL-8 (7). Neurogenic inflammation is mediated by release of neuropeptides such as substance P and calcitonin gene-related protein (8). Furthermore, increased skin sensitivity in patients with rosacea is caused by neurogenic inflammation. Sensitive skin syndrome is also considered a type of SFN (9). Compared to healthy controls, patients with rosacea had higher scores of subjective burning sensations. In contrast, heat–pain thresholds measured by QST were significantly decreased, suggesting C-fiber neurovascular dysfunction (10). Additionally, positive correlation between heat–pain thresholds and clinical severity links rosacea to the SFN spectrum. SFN is also reported to be associated with voltage-gated sodium channel gene variants (11). Abnormal expression of Nav1.8 sodium channel in peripheral nerve axons contributes to hyperpathia in the skin. Regarding rosacea, Nav1.8 expression was significantly upregulated in the epidermis (12). Such findings may represent a potential therapeutic target. IENFD can be measured by immunostaining protein product (PGP9.5); reduction in PGP9.5-positive nerve fibers indicates Aδ, and C-fiber populations are altered. A pathoetiological study has found that the number of immunoreactive fibers in the epidermis of patients with rosacea decreased after laser therapy, which may serve as a contributing neurogenic component to the stinging condition (13). Thus, we hypothesize that PGP9.5-positive nerve fibers may decrease in the complex pathophysiological process of NR, which needs to be further confirmed by direct evidence in the future.

A study conducted in France revealed pruritus as one of the most frequent symptoms in patients with SFN. Sensory symptoms of SFN are often described as length-dependent and stocking-glove distribution; however, non-length-dependent distribution also occurs, frequently involving head-related areas (1). Coincidentally, a considerable proportion of patients with rosacea are accompanied by facial itching sensation, which has an adverse impact on their quality of life (14). Heat and stress, considered to be the most common factors aggravating rosacea, can also worsen pruritus in SFN.

Patients with rosacea frequently present with gastrointestinal symptoms. A nationwide cohort study of the Danish population revealed rosacea is significantly associated with gastrointestinal disorders, such as celiac disease, irritable bowel syndrome, and inflammatory bowel disease; although specific underlying mechanisms remain unclear (15). Patients with SFNs experience gastroparesis, diarrhea, constipation, and intestinal pseudo-obstruction due to C-fiber involvement in the smooth gastrointestinal tract (4). Therefore, it is reasonable to assume that the same pathological process occurs in rosacea. Moreover, cardiac arrhythmias, photophobia, and other symptoms of dysautonomia are observed, which require more attention.

Neurogenic rosacea generally does not respond well to traditional therapy. However, drugs used to treat SFN, such as TCAs, pregabalin, gabapentin, and duloxetine, are effective in treating neurogenic rosacea (16). Furthermore, pulsed dye lasers and endoscopic thoracic sympathectomy have been used to control facial flushing (17).

Notably, there is a lack of case–control studies comparing IENFD in patients with NR to healthy controls, probably for the reason that only a small proportion of rosacea might be NR and also patients tend to refuse the invasive skin biopsy from facial skin for aesthetic reasons. Therefore, more clinical exploration is required. IENFD varies with body regions, suggesting that SFN criteria, such as skin biopsy from the lower leg, is not applicable for facial NR. Nevertheless, NR and SFN have similarity in the pathogenesis, manifestations, complications, and treatment strategies (Table 1).

**Table 1:** Common characteristics between small fiber neuropathy and neurogenic rosacea.

	Mechanism	Clinical symptoms	Assessments	Comorbidity	Treatment
SFN	Damage to the thin Aδ and C-fibers is likely cytokine-mediated *via* TNF-α, IL-2, IL-6, and IL-8	Pain, dysesthesia, dysautonomia and pruritus	Decreased intraepidermal nerve fiber density	Gastroparesis, diarrhea, constipation, and intestinal pseudo- obstruction	TCAs, SNRIs, and anticonvulsants such as gabapentin and pregabalin
NR	Abnormal release of neuropeptides such as SP and CGRP from neuro fibers	Facial burning, stinging, dysesthesia and pruritus	Decreased heat–pain threshold	Coeliac disease, irritable bowel syndrome, inflammatory bowel disease	TCAs, pregabalin, gabapentin and duloxetine

SFN, small fiber neuropathy; NR, neurogenic rosacea; TCA, tricyclic antidepressants; SNRIs, serotonin-norepinephrine reuptake inhibitors; SP, substance P; CGRP, calcitonin gene-related protein.

In conclusion, neurogenic rosacea could be a particular phenotype of SFN that involves facial skin. The current SFN diagnostic criteria are far from perfect, and more attention is required for facial manifestations.
